# Fox’s Atlas and Text-Book of Skin Diseases

**Published:** 1888-06

**Authors:** 


FOX'S ATLAS AND TEXT-BOOK OF SKIN DISEASES. Second edition. Issued in parts by E. B. Treat & Co., New York.

Parts five and six of this noted work continue the consideration of the "inflammatory diseases" of the skin; treating such as eczema, psoriasis, miliaria, lichen, prurigo, herpes, etc. The plates are, as usual, very fine. Price, one dollar each part.

